# Three-way interaction among plants, bacteria, and coleopteran insects

**DOI:** 10.1007/s00425-016-2543-1

**Published:** 2016-05-11

**Authors:** Beata Wielkopolan, Aleksandra Obrępalska-Stęplowska

**Affiliations:** Department of Agrophages’ Forecasting Methods and Agricultural Economic, Institute of Plant Protection, National Research Institute, Poznan, Poland; Interdepartmental Laboratory of Molecular Biology, Institute of Plant Protection, National Research Institute, Poznan, Poland

**Keywords:** Plant–insect interactions, Plant–insect–microbe interactions, Coleoptera, Symbiotic bacteria, Plant response, Protease inhibitors

## Abstract

**Coleoptera, the largest and the most diverse Insecta order, is characterized by multiple adaptations to plant feeding. Insect-associated microorganisms can be important mediators and modulators of interactions between insects and plants.**

Interactions between plants and insects are highly complex and involve multiple factors. There are various defense mechanisms initiated by plants upon attack by herbivorous insects, including the development of morphological structures and the synthesis of toxic secondary metabolites and volatiles. In turn, herbivores have adapted to feeding on plants and further sophisticated adaptations to overcome plant responses may continue to evolve. Herbivorous insects may detoxify toxic phytocompounds, sequester poisonous plant factors, and alter their own overall gene expression pattern. Moreover, insects are associated with microbes, which not only considerably affect insects, but can also modify plant defense responses to the benefit of their host. Plants are also frequently associated with endophytes, which may act as bioinsecticides. Therefore, it is very important to consider the factors influencing the interaction between plants and insects. Herbivorous insects cause considerable damage to global crop production. Coleoptera is the largest and the most diverse order in the class Insecta. In this review, various aspects of the interactions among insects, microbes, and plants are described with a focus on coleopteran species, their bacterial symbionts, and their plant hosts to demonstrate that many factors contribute to the success of coleopteran herbivory.

## Introduction

It is considered that insects represent 60 % of all species on the earth. Herbivorous insects that constitute half of insects (Schoonhoven et al. 1998) are one of the major factors limiting plant growth and fitness. A two-third of all known herbivorous insects species are leaf-eating beetles (Coleoptera) or caterpillars (Lepidoptera) (Schoonhoven et al. 1998; Howe and Jander 2008). Many beetles have beneficial effect on the environment (nutrient recyclers, pollinators), but significant part of them are pests of economically important crops and storage products. Importantly, coleopteran insects cause considerable economic losses to the important staple food crops worldwide: potato, corn, rice, and cereals. For considerable economic losses are responsible among others *Leptinotarsa decemlineata* (Colorado potato beetle, Chrysomelidae), *Oulema melanopus* (cereal leaf beetle, Chrysomelidae), *Diabrotica virgifera virgifera* (western corn rootworm, Chrysomelidae), *Tribolium castaneum* (red flour beetle, Tenebrionidae), *Dicladispa armigera* (rice hispa, Chrysomelidae), *Sitophilus oryzae* (the rice weevil, Curculionidae), and many others.

Plants are exposed to many abiotic and biotic stresses under natural environmental conditions, and it is important that they coordinate the appropriate responses to limit the damage (Voelckel and Baldwin 2004; Stam et al. 2014). Plants are sessile, therefore, effective defense strategies are needed to prevent them from being eaten by herbivorous insects. Plants have a number of defense mechanisms that directly or indirectly affect herbivorous insects. For example, plants are able to enhance their cell walls through lignification (Garcia-Muniz et al. 1998), and synthesize toxic compounds and volatiles (Kessler and Baldwin 2001). Volatiles may also induce defense responses in neighboring plants. A lot of compounds produced by plants are considered as natural insecticides. For instance, plant protease inhibitors (PIs) which belong to the sixth group of pathogenesis-related proteins (PR-6) are considered natural insecticides (Van Loon 1999).

As evidenced by the huge losses in crop yields every year (Jood et al. 1993; Pike and Gould 2002; Tratwal et al. 2014), it is clear that herbivorous insects are able to overcome plant host defenses (Ogendo et al. 2006; Krattiger 1997). Beetles are naturally equipped with anatomical structures to enable them to feed on plants and also have various biochemical and molecular adaptations to overcome plant defense strategies. For example, in response to plant PIs, insects may produce new protease isoforms that are resistant to plant PIs or produce proteases at a higher rate (Shulke and Murdock 1983; Wielkopolan et al. 2015).

In the ongoing interaction between plants and insects, there are ‘hidden’ biotic factors, such as microorganisms associated, both, with plants and insects. These ‘hidden’ factors can significantly influence the plant–insect interaction. Microbes associated with insects may have positive effects on them by aiding in multiple processes, including digestion or protection against pathogens (Dillon and Dillon 2004). In addition, microbes can also modulate plant defense reactions to the benefit of their insects host (Kaiser et al. 2010; Barr et al. 2010). However, microbes associated with plants may also affect the interaction between plants and insects. There is considerable evidence demonstrating that endophytes associated with plants can act as natural insecticides or fungicides (Sturz et al. 1999).

In this review, we focus on plant responses to coleopteran insects as well as the adaptation of those insects to plant feeding and their reactions to plant defense responses. Especially, we would like to emphasis the role of microorganisms associated with herbivorous insects, such as Coleoptera, as the important mediators and modulators of interaction between coleopteran insects and their host plants. We focused on this most numerous insect order not only because of its huge economic importance for agriculture, but also because of its greatest diversity among insect taxa both of which probably are responsible for evolutionary success of Coleoptera. This diversity manifests first of all in the adaptation of Coleoptera to feeding on the wide range of plants (mono- and dicotyledonous), in a variety of niches, which has been continuously expanded starting from pre-Cretaceous period, and in the competition with varying sets of natural enemies. Hence, many articles have been published describing Coleoptera–plant and also Coleoptera–microbe–plant interactions. In this study, we have undertaken to summarize these data indicating also important directions for further studies in this area.

## Economic impact of coleopteran species

Pests belonging to the Coleoptera (the beetles) order are of big interest because of the considerable damages caused by them in the field. The economic impact of widely distributed and harmful chewing insects is described in this part of review.

The order Coleoptera is characterized by the strong screlotized front wings, which protect membranous hindwings (Crowson 1981; Hunt et al. 2007). It is estimated that first beetles appeared around 285 million years ago (Crowson 1981; Grimaldi and Engel 2005). Beetles are characterized by extreme morphological, ecological, and behavioral diversity. Their diversification results most probably from metabolic changes (adaptations to specialized niches and feeding habits) or mutations.

The order Coleoptera includes beneficial insects that may control populations of pests. For example, ladybirds (Coccinellidae) may feed on aphids colonies (Minoretti and Weisser 2000). Ground beetles (Carabidae) are predators of many insects, and may reduce cereal and sugar beet aphids population (Kromp 1999). On the other hand, dung beetle (Scarabidae) improves nutrient recycling and soil structure (Brown et al. 2010). However, many beetles cause huge losses in agricultural production. Among them are leaf-feeding beetles and pests of storage products. It is estimated that worldwide group of storage products pests includes more than 600 species of beetles (Cao et al. 2002; Rajendran 2002). Their infestations may reduce the quality of stored grain, and change the flavor, odor, and color of plant-derived products (Strang and Kigawa 2006). Infestation may prevent grain import what can cause further economic losses (Cao et al. 2002).

**Fig. 1:**
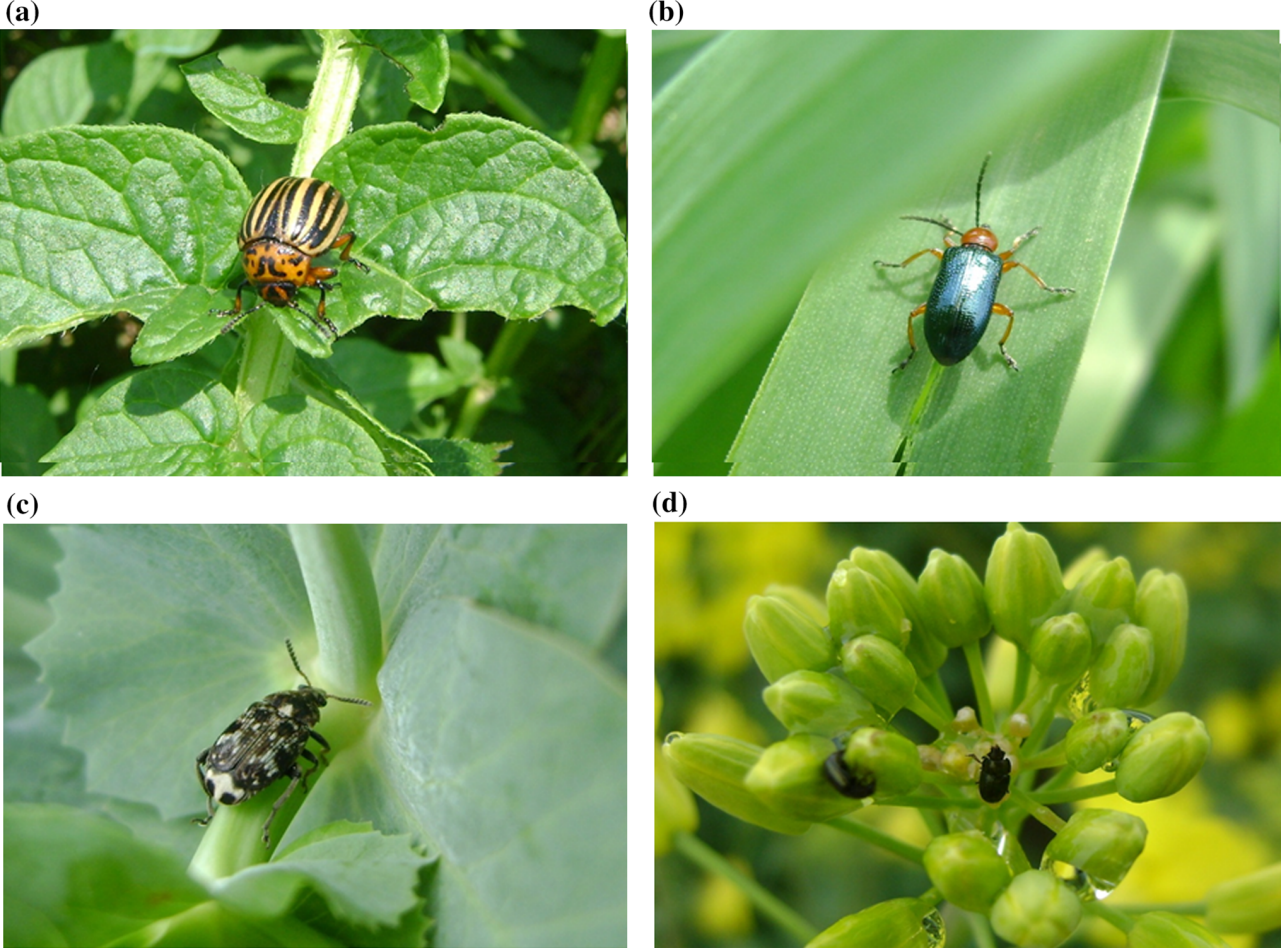
The examples of coleopteran pests of economically important crops belonging to various taxa. **a** *Leptinotarsa decemlineata* on potato (Solanaceae, staple food crop), **b** *Oulema melanopus* on wheat (Poaceae, monocotyledonous plant, staple food crop), **c** *Bruchus pisorum* on pea (Fabaceae, staple food crop), and **d** *Meligethes aeneus* on oilseed rape (Brassicaceae, staple food crop, and plant used for biofuel production)

Crop losses due to pests differ in each country, as they depend on various environmental factors, such as meteorological conditions, prevailing flora, and the types of cultivated crops, as well as the widespread resistance to insecticides. Numerous crop pests, such as the *D. virgifera virgifera* (maize), *L. decemlineata* (potatoes and tomatoes) (Fig. 1a), *O. melanopus* (cereals) (Fig. 1b), *Bruchus pisorum* (pea weevil, Chrysomelidae (pea)) (Fig. 1c), *Meligethes aeneus* (pollen beetle, Nitidulidae) (Fig. 1d), *Tribolium castaneum* or *Trogoderma granarium* (khapra beetle, Dermestidae) (storage products), blister beetles (Meloidae) (Ghoneim 2013), and *Callosobruchus maculatus* (cowpea weevil, Chrysomelidae, stored legumes) are globally distributed. Therefore, there are widespread efforts to strictly control them. However, there are pests that are particularly harmful in specific geographic regions, such as *Ips typographus* (spruce bark beetle, Curculionidae), that is serious pest especially for spruce in Norway and in Eastern Asia, but also in Japan (Christiansen 2008) or *Hypothenemus hampei* (coffee berry borer, Curculionidae), which is reported in the countries with coffee plantations. There are many pests in the Chrysomelidae family alone that are able to damage leaves, roots, seeds, or flowers of susceptible plants. For example, the *D.virgifera virgifera* causes considerable damage to corn fields, especially in the northern USA and Europe. The *D.virgifera virgifera* beetles and larvae are harmful, with the larvae destroying roots and the adult beetles damaging leaves (Levine and Oloumin-Sadeghi 1996). However, larvae are considered to represent the most damaging stage, because their feeding may lead to the decreased ability of roots to transport water and nutrients, resulting in reduced plant growth and grain production (Wright et al. 1999). The larvae and adults of the *L. decemlineata* are responsible for reducing potato crop yields and quality and the resulting negative economic effects. These losses are largely due to the impressive feeding rates of *L. decemlineata* and their high fecundity. In Poland, which is one of the biggest potato-growing countries, an average of 7.1 % of potato plants exhibited *L. decemlineata* feeding symptoms in 2014. However, the damage caused by this pest varied depending on the region with some areas, reporting that 80 % of potato plants were damaged by *L. decemlineata* feeding (Tratwal et al. 2014). An inability to control this pest can lead to the complete destruction of potato fields. Thus, it is very important to control this pest, especially if it has developed resistance to all major classes of insecticides (Alyokhin et al. 2008).

*O. melanopus* is one of the most important cereal pests. The larvae and adult beetles are both capable of significantly damaging cereal leaf tissue, but the larvae cause greater damage. The larvae feed primarily on the first and second leaves (Groll and Wetzel 1984), and cause reduced crop yield and quality. The annual yield losses caused by the *O. melanopus* are considerable with the level of damage depending on location. For example, grain yield losses ranged from 25 % in the US state of Washington (irrigated spring wheat) (Pike and Gould 2002) to 95 % in The Netherlands (Daamen and Stol 1993), and 70 % in central Europe (Dimitrijević et al. 2001).

It is also important to consider *T. granarium*, which is a post-harvest pest of grain and cereal products in many countries. This beetle is believed to be one of the 100 most invasive pests in the world (Lowe et al. 2000). Damages due to the *T. granarium* may be as high as 75 % (Jood et al. 1993).

Another coleopteran insect, *Meligethes aeneus* (pollen beetle, Nitidulidae), is considered a key pest of *Brassica napus* (oilseed rape). In many European countries, such as Denmark, Sweden, Switzerland, and Poland, *M. aeneus* causes losses of up to 60–100 % (Heimbach et al. 2007; Kazachkova 2007; Ahmanl et al. 2009; Breitenmoser 2012; Zamojska et al. 2011). Larvae as well as adult beetles are responsible for these losses (Blight and Smart 1999).

In addition, rice has its own set of herbivorous pests belonging to Coleoptera: *Dicladispa armigera* (rice hispa, Chrysomelidae) and *Sitophilus oryzae* (the rice weevil, Curculionidae) are considered as the most destructive rice pests in Japan (Pathak and Khan 1994). In general, herbivorous insects are believed to be responsible for annual global crop production losses of 20 % (Kerin 1994), whereas global losses caused by insects pests of storage products are estimated to be 25 % of post-harvested grain yield (Cao et al. 2002; Philips and Thorne 2010). In addition, there are numerous other coleopteran insects that are capable of damaging various plant species. Numerous reports underline that multiple coleopteran species are developing insecticide resistance (Chen et al. 2015), which may increase further damages and losses caused by pests.

To summarize above, with high annual financial losses caused by chewing insects, including costs for pest control (for instance in the case of *D.virgifera virgifera* financial losses in Europe are estimated at 472 million Euros annually (Wesseler and Fall 2010)), it is imperative that successful pest management strategies are adopted.

## Insect adaptation to feeding on plants

Host plant quality is very important for many aspects of insect’s life, such as growth and reproduction (Awmack and Leather 2002). However, on the tissue surface occur various morphological structures (e.g., spines, setae, trichomes, thorns, and hairs) which may interfere with insects feeding (Garcia-Muniz et al. 1998). In addition, plant tissue may contain toxic compounds. To overcome these difficulties, insects have evolved many physiological, morphological, and behavioral adaptations that enable feeding, including the type of mouthparts, ways to maintain their grip on plant surfaces during feeding, and detoxification of plant defense compounds. Beetles may be herbivorous scavengers or predators capable of damaging foliage (Chrysomelidae) or seeds (some Curculionidae), and they can also be bark borers (Scolytidae) or nectar feeders (some Buprestidae). The mouthparts of beetles are adapted to biting or chewing. Chewing mouthparts occur in many insect orders, such as Coleoptera (beetles), Lepidoptera (caterpillars), Orthoptera, or Isoptera. Beetle larvae usually have chewing mouthparts, but there may be differences in the feeding habits of larvae and adults. Insects that possess chewing mouthparts are able to create noticeable holes in leaves, wood, or fruits.

Leaf chewers may have adapted to grip exposed leaf surfaces. Their feet usually feature hooks and hairs to help them maintain their grip. Some insects, such as the leaf-feeding beetles (Chrysomelidae), have large toes with pads of hairs on their underside (Beutel and Leschen 2005). In terms of digestion, insects have a wide range of enzymatic activities that facilitate feeding on plants. Included among the enzymes are proteases, which are responsible for breaking down dietary proteins into simple peptides and amino acids (Terra and Ferreira 1994). Proteases are found most abundantly in the midgut region of the insect digestive track, and are subdivided into endopeptidases (proteinases) and exopeptidases. Herbivorous insects have a wide diversity of digestive proteases. It is assumed that insects in the Lepidoptera and Diptera orders generally use serine proteases, while those in the Coleoptera order use cysteine proteases (Murdock et al. 1987). However, it is important to note that each species has its own set of enzymes. In addition, the midgut pH depends on the species and provides the optimal condition for protease activity. Serine proteases require alkaline conditions, whereas cysteine proteases function best in an acidic environment. Aspartyl proteases often occur together with cysteine proteases, as is the case in *Hypera postica* (alfalfa weevil, Curculionidae) (Wilhite et al. 2000). Cysteine proteases were found in the following coleopteran families: Meloidae, Coccinellidae (*Epilachna varivestis*, Mexican bean beetle) (Murdock et al. 1987), Tenebrionidae (*T. castaneum*) (Murdock et al. 1987), Bruchidae (*Zabrotes subfasciatus*, Mexican bean weevil) (Lemos et al. 1987), Chrysomelidae (*C. maculatus* and *Acanthoscelides obtectus*, bean weevil) (Kitch and Murdock 1986; Campos et al. 1989; Wieman and Nielsen 1988), Curculionidae, and Silphidae (Terra and Cristofoletti 1996). Serine protease activities were observed in *T. granarium* (Hosseininaveh et al. 2007) and *Rhynchophorus ferrugineus* (red palm weevil, Curculionidae) (Hernández et al. 2003).

Insects can also efficiently use both serine and cysteine proteases to digest proteins because of the compartmentalization of protease activities to the posterior and anterior portions of the midgut, which have different pH levels (Thie and Houseman 1990). For example, in *Tribolium molitor* (mealworm beetle, Tenebrionidae) larvae, the pH in the anterior midgut is 5.9, whereas in the posterior region, it is 7.9. The proteases are located in the regions with the optimal pH for activity. This compartmentalization of enzyme activities also occurs in *T. castaneum* larvae (Oppert et al. 2005). The presence of three mechanistic classes of proteases (i.e., cysteine, serine, and aspartyl proteases) was reported in *Lissorhoptrus brevirostris* (rice water weevil, Curculionidae) (Hernández et al. 2003), while four classes were observed in *Oulema* spp. larvae (Wielkopolan et al. 2015). Taking all of afore-mentioned data into account, it can be concluded that beetles are relatively similar in terms of morphological and physiological adaptations enabling feeding (mouthpart, basic organization of the digestive tract). However, the insect digestive profile (enzymes content and optimal conditions of their activities) can be very diverse. This diversity reflects beetles’ adaptations to specialized niches and feeding habits. Importantly, insects digestive systems are not passive, but are able to adapt to plant toxins and antinutritional compounds.

**Fig. 2:**
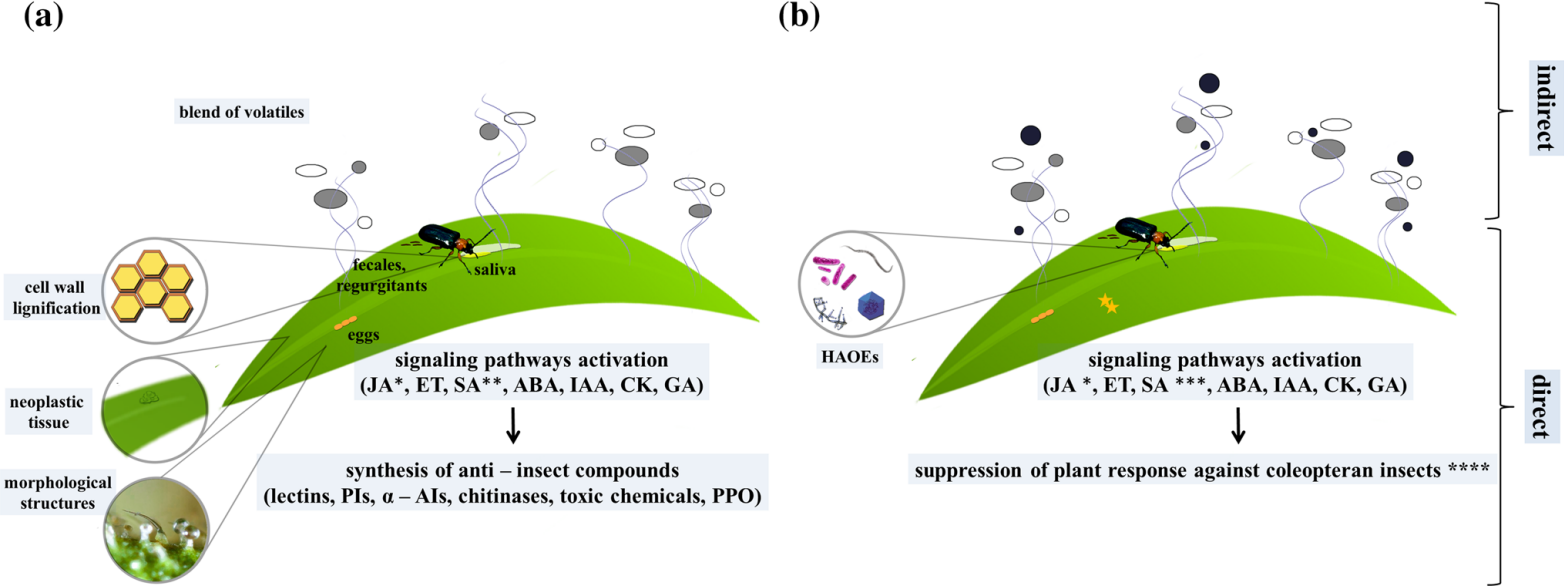
Proposed scheme of plant direct and indirect responses against insects and influence of microbial factors on plant–insect interaction. **a** Plant direct response includes: morphological structures on the leaf surface (e.g. spines, setae, trichomes, thorns, and hairs) that may interfere with insects feeding, strengthening of the cell wall through lignification (tissue is less palatable to herbivores what inhibits insect feeding), and formation of neoplastic tissue (which inhibits pest entry into the plant). Response occurs not only in the damaged place, but the signal is transmitted to other parts of plant. Plant indirect response is associated with volatile substances emission. In response to insect feeding jasmonic acid (JA)-/ethylene (ET)-dependent pathways are activated, and thus, downstream defense response is initiated, including synthesis of antinutritional proteins [e.g. lectins, protease inhibitors (PIs), and alpha-amylase inhibitors (α-AIs)]. **b** An important role in the plant–insect interaction play ‘hidden’ factors, such as microbes associated both with insects and plants. Plant-associated bacteria are localized either in the rhizosphere or in the phyllosphere (*stars*). These bacteria may interfere with plant signaling pathways which may have positive or negative effect on insect fitness. In addition, some plant-associated microbes may produce toxins that act as bioinsecticides. On the other hand, beetles-associated organisms and elicitors having contact with plant tissue during insect feeding act as modulators and modificators of plant defense response to the benefits of their insect hosts. For instance, microbes may modify plant response what leads to alterations in plant volatiles composition (*circles*) and defense-related molecules expression. Insects-associated microbes may shift plant response from JA-dependent to salicylic acid (SA)-dependent pathway. *Asterisk* JA is considered as the most important regulators in plant defense against insects (Watanbae et al. 2001; Howe and Jander 2008; Pieterse et al. 2012). *Double asterisk* SA is less important in plant response to chewing insects. *Triple asterisk* degree of SA involvement in plant response is dependent on the composition of insect-associated bacteria. *Four asterisk* bacteria contribution in the suppression of the plant response against Coleoptera was proposed in research papers (including Barr et al. 2010; Chung et al. 2013). *ABA* abscisic acid, *IAA* indole-3-acetic acid, *GA* gibberellic acid, *CK* cytokinin, *HAOEs* herbivore-associated organisms and elicitors, *PPO* polyphenol oxidase

The oral secretions of insects consist of a mixture of components that allow for feeding on plant material. Herbivorous pests are associated with various organisms and elicitors (HAOEs—herbivore-associated organisms and elicitors; Zhu et al. 2014; Bonaventure et al. 2011) that function during insect feeding. The oral secretions are diverse and may include enzymes (glucose oxidase and β-glucosidase) (Mattiacci et al. 1995; Eichenseer et al. 1999), modified forms of lipids [fatty acid and amino acid conjugates and sulfur-containing fatty acids (caeliferins)] (Alborn et al. 2007; Hilker and Meiners 2010), cell-wall fragments (pectins and oligogalacturonides) (Bergey et al. 1999), peptides from digested plant proteins (Schmelz et al. 2006), or organisms (microbes, fungi, viruses, and parasites), and/or organism-derived proteins (Hughes et al. 2012) that interfere with the outcome of the plant–insect interaction. The insect elicitors are not considered as general elicitors, because they are usually restricted to a specific plant–insect interaction. Some herbivores may have effector molecules that can suppress plant defense responses (Walling 2009). In most cases, the effector molecules suppress a jasmonic acid (JA)-dependent pathway, which is mostly activated in response to herbivorous insects (Chung et al. 2013). These effector molecules may be present in insect oral secretions or eggs (Consales et al. 2012; Atamian et al. 2013) (Fig. 2). For example, *L. decemlineata* harbours multiple bacteria symbionts in oral secretion that can be transferred to the plant during feeding. Flagellin derived from *Pseudomonas* sp. induces salicylic acid (SA)-dependent pathway and suppress JA signaling pathway (cross-talk), what consequently reduces plant defense against the beetles (Chung et al. 2013).

Insects may also be associated with microbes that are pathogenic for plant. These plant pathogens not only may suppress plant response to the benefit of their insect host but may also change plant architecture and/or physiology to attract vectoring insects to increase the chances of pathogens’ dispersal (Bai et al. 2009).

In conclusion, insects are not simple, but constitute very complex organisms community capable of flexible adaptations to the prevailing challenges to which insect host is exposed. Therefore, the future studies should be aimed at characterization of the compositions of particular insect communities as well as search for a factor or factors disturbing insect physiology as well as explaining their roles in plant–insect interactions.

## Plant defense strategies

Plants respond to herbivores attack either directly or indirectly (Arimura et al. 2009) (Fig. 2). Direct plant responses inhibit insect processes, such as reproduction or digestion, while also contributing to improved mechanical protection on plant surfaces (e.g., spines, setae, trichomes, thorns, and hairs). The plant cell wall is considered the first line of defense. In response to an attack by herbivorous insects, the cell wall is strengthened through a lignification process, which makes tissue less palatable to herbivores and inhibits insect feeding (Garcia-Muniz et al. 1998) (Fig. 2a). These plant responses ultimately disturb the biological activities of the attacking insects, thereby leading to some protection from damage. Plants produce chemicals (e.g., terpenoids, alkaloids, anthocyanins, phenols, quinones, flavones, and isoflavones) (Hanley et al. 2007; Engelberth 2006) or proteins (e.g., PR proteins) that are toxic to insects. Ryan (2000) categorized plant proteins newly synthesized after wounding into three groups: (1) antinutritional proteins or defensive proteins (e.g., PIs) or proteins involved in secondary compound biosynthesis, (2) signaling pathway proteins, and (3) proteins involved in rerouting metabolic activities to the production of defensive compounds, such as proteases. Some plants are able to accumulate and store toxic compounds to ensure an immediate response to attacking herbivorous insects. Plants that do not accumulate defensive compounds may minimize damage through rapid growth (Jander et al. 2001).

A unique plant response to coleopteran insect feeding may involve the formation of neoplasmic tissue that impedes larval entry into the plant host (Doss et al. 2000) (Fig. 2a). In addition, during oviposition, some elicitors that may influence plant responses are produced. For example, fatty acids, such as bruchins, which are α,ω-diols esterified at one or both oxygens with 3-hydroxypropanoic acid, derived from *B. pisorum* and *C. maculatus* are considered potential regulators of neoplastic growth of pea pods. In addition, bruchin B can up-regulate the expression of *CYP93C18*, leading to an increased production of pisatin and isoflavone phytoalexin, which are involved in plant defense mechanisms (Cooper et al. 2005). Furthermore, callus formation inhibits larval entry into the pods (Doss et al. 2000). Plants protect themselves against biotic and abiotic stresses with a highly sophisticated network of signal transduction pathways, which are regulated by different hormones (Pieterse et al. 2012). Phytohormones may also affect plant interactions with beneficial organisms, such as microbes (Gutjahr and Paszkowski 2009; Hause and Schaarschmidt 2009).

Plant responses can be categorized as systemic acquired resistance (SAR) or induced systemic resistance (ISR). In general, ISR is associated with defense against pests, and may be induced by nonpathogenic bacteria, abiotic factors or feeding by herbivorous insects (Watanabe et al. 2001; Galzebrook 2005; Howe and Jander 2008). ISR is associated with signaling pathways dependent on jasmonic acid (JA) or ethylene (ET). In SAR, plants are protected against infection by a wide range of pathogens. The activation of SAR requires endogenous salicylic acid (SA) and its functional metabolites. SA is associated with plant defense against biothropic pathogens (Glazebrook 2005) and phloem-feeding herbivores (Kaloshian and Walling 2005). Importantly, SA involvement is believed to be greater in plant response against piercing and sucking type of insects pests than the chewing insects (War et al. 2012; Zhao et al. 1996). JA, SA (Pieterse et al. 2012), and ET (Adie et al. 2007) are considered as the fundamental regulators of plant defense response against attackers (Pieterse et al. 2012). The main role of hormones is a reprogramming of plant genetic machinery that leads to the adequate plant response to external stressors. Interaction between individual components of a highly sophisticated network of signal transduction pathways can be additive, antagonistic, or synergistic. Ethylene pathway is activated, likewise JA-mediated pathway, in response to necrotrophic pathogens and often works synergistically with JA (Chen et al. 2005; Von Dahl and Baldwin 2007). It is considered that JA and SA are effective against different groups of insects and pathogens. The cross-talk between these two main signaling pathways (SA, JA) allows plants to fine-tune defense responses (Thaler et al. 2012). In general, it is considered that SA acts antagonistically to the JA-pathway (Spoel et al. 2003). This trade-off can occur when plant is attacked simultaneously by various pathogens (Koornneefer et al. 2008). Others plant phytohormones, such as abscisic acid (ABA), auxins [indole-3-acetic acid (IAA)], cytokinin (CK), or gibberellic acid (GA) (Robert-Seilaniantz et al. 2011; Torres-Vera et al. 2014) act as secondary players and modulators of main signaling pathways. For instance, ABA has a primary role in the regulation of plant defenses against abiotic stressors. It may also play a role in plant responses against pathogens (Beattie 2011; Ton et al. 2009) or herbivores (Erb et al. 2009; Verhage 2011), as it may affect multiple signaling pathways.

Herbivorous insects, including coleopteran ones, can evade plant response through employing some factors, such as bacteria, obligate pathogens that are able to suppress JA-dependent defenses. In effect, plant recognizes beetles as microbes and is not able to induce effective response against these insects (Chung et al. 2013).

After induction of defense signaling pathways, the plant is able to synthesize the group of antinutritional proteins that can reduce the ability of insects to digest plant material. This group of antinutritional proteins belong to , e.g, protease inhibitors (PIs), alpha-amylase inhibitors (α-AIs), lectins, chitinases, and polyphenol oxidases (PPO) (Fig. 2a). The up-regulation of these proteins was frequently observed during Coleopetera–plant interactions as stated below.

Agglutinin and arcelin, which are lectins (sugar-binding proteins) from *Phaseolus vulgaris*, are toxic to *C. maculatus* (Gatehouse and Gatehouse 1998) and *Z. subfasciatus*, respectively (Osborn et al. 1988). In addition, *Talisia esculenta* (Sapindaceae) lectins showed anti-insecticidal activity against *C. maculatus* and *Z. subfasciatu*s larvae (90 % mortality). Allsopp and McGhie (1996) reported that snowdrop and wheat germ lectins can suppress the growth of *Antitrogus parvulus* (sugarcane white grub, Scarabaeidae) larvae. Agglutinin from wheat germ inhibits larval growth of *Diabrotica undecimpunctata howardi* (southern corn rootworm, Chrysomelidae) (Czapla and Lang 1990). Canatoxin isolated from *Canavalia ensiformis* (jack bean, Fabaceae) had a toxic and lethal activity against insects with cathepsin-based digestion. It caused complete inhibition of *C. maculatus* larval growth (Carlini et al. 1997).

PR proteins warrant particular attention, especially the PIs of PR-6. The PIs naturally occur in plant leaves and storage organs and their abundance significantly increases in response to wounding (Sharma 2015), which suggests their important roles in plant defense. The PIs help to regulate plant protease activity affecting plant developmental processes, such as programmed cell death (Pernas et al. 1999) or protein mobilization in storage tissue. It is important to note that PIs are considered effective against pests, because they inhibit digestive proteases in the insect gut. The disruption of digestive processes negatively influences insect growth and development. The PIs can also affect a number of other vital processes, such as proteolytic activation of enzymes and molting (Sharma 2015). For example, the gene encoding the cysteine PI, oryzacystatin, which inhibits cysteine proteases in the digestive track of *Chrysomela tremulae* (poplar leaf beetle, Chrysomelidae), was transformed into transgenic poplar plants. Feeding tests indicated that the transgenic plants highly expressing oryzacystatin were toxic to *C. tremulae* larvae (Leplé et al. 1995). On the other hand, the trypsin-papain inhibitor PdKI2 of *Pithecellobium dumosum* (Fabaceae) seeds effectively inhibited the digestive proteases of the bruchids *Z. subfasciatus* and *C. maculatus* (Oliveira et al. 2007). The proteinaceous Kunitz-type trypsin inhibitor from *Crotalaria pallida* (Fabaceae) seeds, CpaTI, inhibited the digestive enzymes of *Z. subfasciatus*, *C. maculatus*, and *Anthonomus grandis* (boll weevil, Curculionidae) to varying degrees (Gomes et al. 2005). A serine PI from *Amaranthus hypochondriacus* (Amaranthaceae) actively suppressed the proteolytic activity of chymotrypsin and trypsin from *Prostephanus truncatus* (larger grain borer, Bostrichidae) (Houseman and Thie 1993).

In plant defense responses, α-AIs, which are plant PR proteins, also play important roles. Wheat α-AIs may inhibit α-amylase enzymes in *Tenebrio obscurus* (mealworm, Tenebrionidae), *Tribolium* spp. (flour beetle, Tenebrionidae), *Sitophilus* spp. (wheat weevils, Curculionidae), and *Oryzaephilus* spp. (grain beetle, Silvanidae). In addition, α-AIs protect transgenic peas from *B. pisorum* (Morton et al. 2000).

On the other hand, chitinases that also belong to PR proteins, digest chitin which is a component of insect exoskeletons and peritrophic membranes (Kramer et al. 1997). Transgenic *Solanum lycopersicum* (Solanaceae) overexpressing the WIN6 chitinase was observed to be resistant to *L. decemlineata* attack (Lawrence and Novak 2006).

**Fig. 3:**
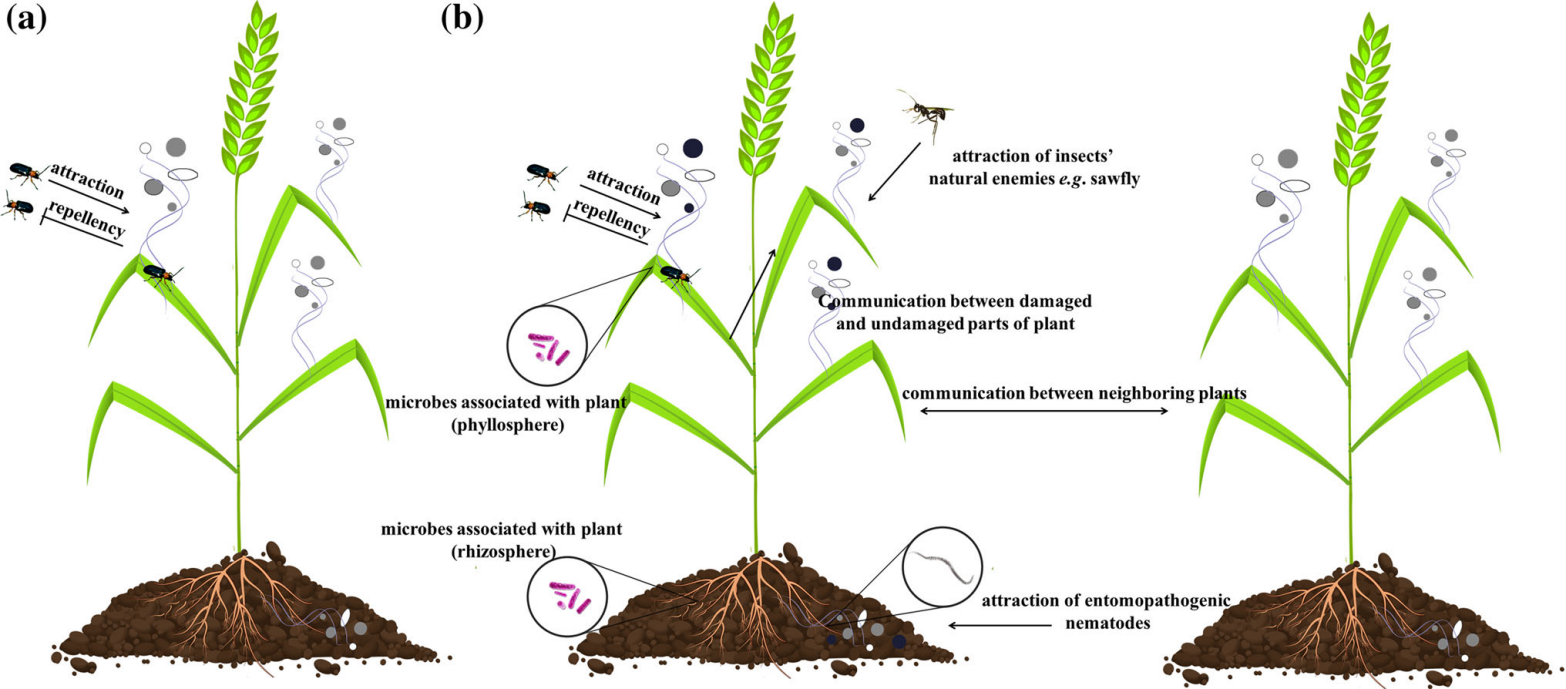
Volatiles emission during plant–insect interactions. **a** Plant releases the blend of volatiles (*different colored circles*) that may repel plant pests and attract beneficial insects (e.g. pollinators). However, some plant pests are also attracted by plant volatiles. **b** Plants are able to recognize differences between mechanical wounding and insects feeding what results in a different composition of volatiles compounds. The plant, wounded by insect feeding, may emit volatiles which attract pests’ natural enemies (parasites, predators, including entomopathogenic nematodes), repel herbivorous insects (including Coleoptera), induce defense responses in neighboring plants as well as function in the communication between damaged and undamaged parts of plant. In addition, microbes associated both with plants (*enlarged circles* in the rhizosphere and phyllosphere) and insects may modulate plant volatiles composition. Moreover, insect-associated pathogens of plants may modulate plant physiology to attract their potential insect vectors

Plant defense responses to insect feeding occur not only at or near the site of damage, but also throughout the plant because of signaling molecule-based communication between different plant parts (Fig. 3). A systemic and local response may result in the production of the same defensive proteins, but there may be differences in the kinetics of their production. For example, PIs are produced because of induced defense responses, but may also accumulate as part of constitutive defense responses. Phytoecdysteroids (defense compounds) accumulate in *Spinacia oleracea* (spinach, Amaranthaceae) foliage and their synthesis is up-regulated in response to tissue damage caused by *O. sulcatus* (Schmelz et al. 1999). Similarly, there is an increase in glucosinolate content in response to feeding by *Psylliodes chrysocephala* (cabbage stem flea beetle, Chrysomelidae) (Bartlet et al. 1999). Therefore, plant defense compounds accumulate before insect feeding, and herbivory induces the synthesis of these compounds at a higher rate (Garcia-Olmedo et al. 1987; van Dam et al. 2001).

Indirect responses to insects are mediated through the release of a mixture of volatiles, which may attract predatory and parasitic insects that are natural enemies of herbivores (De Moraes et al. 2001; Dicke et al. 2003), repel herbivores (Kessler and Baldwin 2001), induce defense responses in neighboring plants or function in the communication between damaged and undamaged parts of a plant (Karban et al. 2000; Engelberth et al. 2004) (Fig. 3). Plant volatile emission can be, however, a double sword, because they also attract plant pests which feed on these plants. The release of volatiles may have some detrimental effects for plants. There is evidence showing that certain inducible plant volatiles can attract coleopteran insect pests. For example, the *L. decemlineata* is attracted to plants by a mix of volatiles and methyl jasmonate (Dickens 2006). Volatiles released by *Ipomoea batatas* (sweet potato) attract *Cylas formicarius* (sweet potato weevil, Curculionidae) (Korada et al. 2010). Moreover, von Mérey et al. (2011) observed that *D. virgifera virgifera* beetles occur more frequently in fields treated with green leaf volatiles, which suggests the volatiles have a role in attracting the beetles. It was also observed that the beetles prefer the leaves of *Vitis labrusca* and *Malus* spp. infested by *Popillia japonica* (Japanese beetle, Scarabaeidae) over undamaged leaves (Loughrin et al. 1995, 1996). Plant volatiles may also mediate the interaction among plants, insects, and microbes (Dicke and Baldwin 2010). They are released in large amounts during attacks by herbivores (Turlings et al. 1995; Tumlinson et al. 1999). Noge et al. (2011) reported the emission of plant volatiles [phenylacetonitrile, (E)-β-ocimene, linalool, (E)-4,8-dimethyl-1,3,7-nonatriene, and (E,E)-α-farnesene] from the leaves of *Fallopia sachalinensis* (giant knotweed plants) during a *P*. *japonica* attack. Interestingly, in the case of this insect, plant volatiles were not emitted from either undamaged leaves or leaves that were mechanically wounded. These results suggest that the synthesis of volatiles is induced de novo in *F. sachalinensis* by an elicitor contained in the oral secretions of *P. japonica*. Noticeably, it is considered that terpenoids, such as (E)-β-farnesene, play a key role to the attraction natural enemies of insects. (E)-β-farnesene is considered as principal component of the alarm pheromone of many aphid species (sap-sucking insects) (Beale et al. 2006; Al Abassi et al. 2000; Pickett et al. 1992). In general, it is assumed that plants are able to differentiate between herbivore damage and wound response by emission of certain types of volatile substances during feeding, which are not present during only mechanical wounding (Delphia et al. 2006) (Fig. 3b). Volatiles emission and their attraction of pest’s natural enemies were also studied for other beetles. For example, *Zea mays* roots attacked by *D. virgifera virgifera* larvae release the sesquiterpene (E)-β-caryophyllene, which attracts *Heterorhabditis megidis* entomopathogenic nematodes that feed on the larvae (Rasmann et al. 2005). Genetically modified maize plants that constitutively produce (E)-caryophyllene attract nematodes more effectively than wild-type controls, resulting in reduced root damage caused by *D. virgifera virgifera* larvae (Degenhardt et al. 2009). Similarly, when *Thuja occidentalis* is attacked by *Otiorhynchus sulcatus* (black vine weevil, Curculionidae), it releases volatiles from the roots, which also attract the entomopathogenic nematode *H. megidis* (van Tol et al. 2001).

In conclusion, plants are able to recognize mechanical wounding from damage caused by insect feeding and produce plant volatiles of different compositions. Plants are sessile and as such they are in a worse position, because they can not escape from insects, especially so well adapted to feeding as Coleoptera. Nonetheless, plants have developed a series of defense mechanisms allowing them to (a) defend themselves (a series of defense events, from recognition to attack), (b) to warn their neighbors against danger (releasing a blend of volatiles), (c) to attract insect natural enemies. As mentioned above insects have adopted to diverse plant defense mechanisms. On the other hand, plants also developed various adaptations to insects attack what further resulted in the genetic variation of insects pests. What is noticeable, plants likewise insects have hidden players-microorganisms that may have a considerable impact on the outcome of this ongoing plant–insect battle which will be discussed below.

Based on the current knowledge we can deduce that plant volatiles may be used to develop new, environmentally friendly strategies for crop protection in the future. First, volatiles may be used to enhance the attractiveness of crop plants to biological control agents what was confirmed by field studies (e.g. Degenhardt et al. 2009). Secondly, they may be used to develop trap crops (attraction of pests). Therefore, the knowledge of the plant volatiles composition is very important as well as the analysis of the possibility of plant volatiles application as effective method of limiting pest harmfulness and thus economic losses.

## Insect reactions to plant defense

During feeding, insects may consume harmful substances, such as plant defense compounds. Plant-derived toxins may have a broad range of activities and exhibit highly diverse molecular structures and physical properties. The concentration of these compounds depends on the organs in which they are produced and the plant developmental stage (Gebrehiwot and Beuselinck 2001). Plant defense compounds have forced herbivores to evolve strategies that enable them to recognize and avoid these compounds to prevent ingestion of lethal doses. These strategies can be genetically determined, inherited, or learned (Chapman 2003; Després et al. 2007; Schowalter 2011). Coleopteran insects may avoid the effects of plant toxins through behavioral, physical, and biochemical mechanisms, including the production of detoxification enzymes, such as esterases, glutathione-*S*-transferases, and cytochrome P450 monooxidases (Li et al. 2007). They may also adapt to the toxic compounds or avoid ingestion of toxic substances by feeding on non-toxic plant organs or during developmental stages where toxins are absent (Hoy et al. 1998; Després et al. 2007). Insects may also feed on different plant hosts to avoid lethal doses of plant defense compounds (Pankoke et al. 2012). There are numerous reports showing how coleopteran insects deal with plant toxic substances. For example, the specialist beetle *E. varivestis* has reduced endogenous β-glucosidase activities compared with the generalist locusts (grasshopper, Acrididae). During feeding, the beetles hydrolyze more cyanogenic glucosides than the locusts because of differences in how the insects feed. Beetles (leaf-chewing) have relatively small mandibles that force them to chew leaves and crush plant tissue, but locusts (leaf-snipping) have larger mandibles that allow them to consume larger leaf pieces, resulting in a higher percentage of plant tissue being ingested and more limited hydrolysis of cyanogenic glucosides (Ballhorn et al. 2010). Insects may sequester toxic plant compounds. Some plants (crucifer plants, Brassicales) are equipped with the glucosinolate–myrosinase (“mustard-oil bomb”) defensive system which is activated during insect attack. Some beetles, such as *Phyllotreta striolata* (striped flea beetle, Chrysomelidae) avoid this system throughout selective accumulation of substrate (glucosinolate) that is activated by their own myrosinase (Beran et al. 2014). Some insects are able to consume and accumulate plant defense compounds in their tissues, such as the hemolymph or defense glands (Nishida 2002; Optiz and Muller 2009). Insects that sequester toxic phytocompounds may be toxic to their own predators (Discher et al. 2009). For example, leaf beetles, such as *Chrysomela populi* (broad-shoulder leaf beetles, Chrysomelidae) and *Phratora vitellinae* (brassy willow beetle, Chrysomelidae) sequester the salicinoid salicin from *Salix* spp. (Salicaceae) and transport it from the gut to the hemolymph and finally to the defense glands (Kuhn et al. 2004; Burse et al. 2009). β-Glucosidases hydrolyze the salicin to saligenin, which acts as a deterrent to predators (Kuhn et al. 2004; Optiz and Muller 2009). Additionally, *Chrysomela lapponica* (leaf beetle, Chrysomelidae) larvae that feed on plants from the Salicaceae family (e.g., willow and poplar trees) sequester plant-derived salicin and other leaf alcohol glucosides, which accumulate in their defensive glands and are modified to bioactive compounds (Burse et al. 2009).

The detrimental effects of inhibitors on insects have been well documented. The negative effect of cysteine PIs on the growth of certain coleopteran species was shown years ago (Orr et al. 1994). The *L. decemlineata* uses cysteine and aspartyl proteases (Michaud et al. 1993). As demonstrated using the synthetic inhibitor E-64 (*trans*-epoxysuccinyl-l-leucylamido(4-guanidino)butane), cysteine PIs significantly inhibit *L. decemlineata* larvae growth (Wolfson and Murdock 1987). Additionally, cysteine PIs have been shown to affect the protease activity of coleopteran larvae, such as those of *D. undecimpunctata howardi* (Fabrick et al. 2002) or the *D. virgifera virgifera* (Zhao et al. 1996). Generally, pests have evolved different adaptations to reduce the harmful activities of PIs. They may increase digestive enzyme activity, synthesize more resistant proteases (Paulillo et al. 2000), digest inhibitors in the gut (Girard et al. 1998), decrease the sensitivity of their enzymes to inhibitors (Brito et al. 2001). For example, proteases of *Z. subfasciatus* are capable of degrading an α-AI from the common bean (Ishimoto et al. 1996). The soybean cysteine PI soyacystatin N (scN) is capable of suppressing the digestive enzymes of herbivorous insects and can inhibit the growth and development of *C. maculatus*, *L. decemlineata*, and *D. virgifera virgifera* (Zhao et al. 1996; Koiwa et al. 1997; Zhu-Salzman et al. 2003). *C. maculatus* has evolved counter-defensive strategies against scN, such as increasing the expression of scN-sensitive and scN-insensitive enzymes and hydrolyzing scN (Zhu-Salzman et al. 2003). Oppert et al. (2004) reported that *T. castaneum* larvae have evolved mechanisms to overcome dietary inhibitors. Although larvae of this pest produce cysteine and serine proteases, cysteine proteases are the major digestive proteases. Serine and cysteine PIs alone had minimal effects on larvae development and protease activity because the digestive preferences were switched from cysteine protease-based to serine protease-based digestion. Larval growth was inhibited when both cysteine and serine PIs were present. Additionally, Zhu-Salzman et al. (2003) indicated that *T. castaneum* responds to cysteine PIs by increasing the production of aspartic proteases. However, the *L. decemlineata* responded to cathepsin D inhibitors in transgenic plants by decreasing the production of inhibitor-sensitive enzymes (Brunelle et al. 2004). Further, in *Oulema* spp. larvae that were fed the synthetic serine PI AEBSF (4-(2-aminoethyl)benzenesulfonyl fluoride hydrochloride), two additional protease activities were observed (Wielkopolan et al. 2015).

Interestingly beetles may also use proteases of endosymbiotic bacteria inhabiting their gut, what can lead to the change of insect’s food preferences (adaptation of insect to a new host plants) (Chu et al. 2013; Shao et al. 2012). For instance, in this way *D. virgifera virgifera* adapted to feeding on the non-host plants, such as soybean (*Glycine max*), which was introduced into the corn field for crop rotation (Chu et al. 2013).

Presented examples of beetles adaptation to inhibitory or toxic plant compounds showed that when the insects were exposed to one class of PIs, they shift to the production of a different class of proteases. When more than one class of PIs was present, then the larvae were unable to adapt using another class of proteases. As mentioned above insects digestive system is not passive but flexible. Profile of insect’s digestive enzymes may undergo changes in response to plant anti-feeding substances (e.g. PIs). To all these afore-mentioned adaptations of beetles considerably contribute insect-associated microorganisms.

## Insects as a well-organized community

Insects harbor for a large array of microbes so they cannot be considered as individuals but as a community. The microorganisms inhabiting the insect gut may include viruses, parasitoid larvae, bacteria, parasitic worms, and fungi (Hughes et al. 2012). Insect-associated organisms not only affect reproduction, digestion, morphology, and behavior, they may also modify plant defense mechanisms for the benefit of their insect host. As mentioned above gut microorganisms can also significantly affect insect evolution by influencing adaptations to specialized niches and feeding habits.

Fungi are frequently observed in the guts of insects that feed on wood or detritus, and are believed to be involved in digestion. For example, many subcortical insects, such as bark beetles (Curculionidae) have fungal symbionts that confer a variety of benefits to the insect (Douglas 2009). In *Anoplophora glabripennis* (Asian longhorned beetle, Cerambycidae), lignin degradation may occur primarily because of fungal activities (Geib 2008). However, in this review, we focus only on coleopteran insect-associated bacteria.

Studies have revealed that the bacteria inhabiting the insect gut are largely nonpathogenic and in most cases positively affect the insect host. They may affect digestion (Koga and Tsuchida 2003), reproduction (White et al. 2009), defense against natural enemies (e.g., predators and parasites) (Oliver et al. 2010), or genetic differentiation (Charlat et al. 2009). They may also function as elicitors or effectors and modify interactions between plants and insects to favor the insect host. There are a variety of bacterial phyla represented in the insect gut, including: *Gammaproteobacteria*, *Alphaproteobacteria*, *Betaproteobacteria*, *Bacteroidetes*, *Firmicutes* (*Lactobacillus* and *Bacillus*), *Clostridia*, *Actinomycetes*, *Spirochetes*, *Verrucomicrobia*, and *Actinobacteria* (Colman et al. 2012). However, each insect species has its own set of associated organisms, which is influenced by the secondary compounds consumed in the diet (Kohl and Dearing 2012) and this diet is extremely diverse in the case of beetle species. For example, beetles of *D. virgifera virgifera* are associated with endosymbiotic *Wolbachia* spp. and enterobacteria (Barr et al. 2010). *Wolbachia* spp. are present intracellularly throughout the insect body, including in the salivary glands and reproductive tissue, where they are found at high concentrations. It is estimated that *Wolbachia* can be associated with 20–70 % of all insects species (Jeyaprakash and Hoy 2000; Zug and Hammerstein 2012). It has been reported that *Wolbachia* may protect the host from pathogens (Eleftherianos et al. 2013), restore or affect fertility or overcome plant defense response (Starr and Cline 2002) (Barr et al. 2010). For example infection of *T. castaneum* with *Wolbachia* causes cytoplasmic incompatibility and reduced fertility of infected *T. castaneum* females was observed (Wade and Chang 1995). In addition, females of *T. castaneum* without bacteria *Wolbachia* lay sterile eggs although they were mated with infected males (Wade and Stevens 1985). The larvae of *L. decemlineata* can be associated with symbionts belonging to the genera *Stenotrophomonas*, *Pseudomonas*, and *Enterobacter* (Chung et al. 2013) as well as with *Flavobacterium* endosymbionts (Krawczyk et al. 2015). Symbionts inhabiting the insect gut can be vertically transmitted. For example, microbes present in the cytosol of the foregut cells of grain weevil larvae (Sitophilus) migrate to the midgut epithelial cells in adults (Dale et al. 2002). The symbiont of *Macroplea appendiculata* and *M. mutica* (reed beetles, Chrysomelidae) is also vertically transmitted (Kölsch et al. 2009). The abundance of bacteria inhabiting the insect gut is affected by pH or the production of enzymes, including lysozymes, such as peptidoglycan hydrolases, which digest bacterial cells (Dubreuil et al. 2001). Some insects are able to control symbionts because of the presence of antimicrobial peptides. For example, *Sitophilus zeamais* (maize weevil, Curculionidae) uses the antimicrobial peptide coleoptericin A to inhibit endosymbiont cytokinesis by limiting bacterial cell division and dispersion (Login and Heddi 2012). Microbes associated with herbivorous insects can also protect their host against fungal species. Based on the results of controlled assays, microbes in the oral secretions of *Dendroctonus rufipennis* (spruce beetle, Scolytinae) were observed to inhibit the growth of fungal species responsible for reducing spruce beetle reproduction and survival (Cardoza et al. 2006).

Insect gut microorganisms may also be involved in the detoxification of food. Some sources of nutrients are available only if the associated toxins can be neutralized. Insect-associated microbes can metabolize insecticides (Kikuchi et al. 2012), heavy metals (Senderovich and Halpern 2013), and plant defense chemicals (Boone et al. 2013; De Fine Licht et al. 2013; Hammerbacher et al. 2013). For example, symbiotic yeast in the gut of *Lasioderma serricorne* (cigarette beetle, Anobiidae) can degrade dietary toxins and increase host resistance (Dowd and Shen 1990). In large numbers, *Dendroctonus ponderosae* (mountain pine beetle, Curculionidae) can kill healthy conifers (Blomquist et al. 2010) even though the trees may possess toxic compounds, such as monoterpenes and diterpene acids (Raffa et al. 2005). Boone et al. (2013) reported that bacteria (*Serratia*, *Pseudomonas*, *Rahnella*, and *Brevundimonas*) associated with *D. ponderosae* are able to metabolize monoterpenes and diterpene acids. For instance *Serratia* reduced concentration of all monoterpenes applied to media by 55–75 % (except α-pinene).

Interestingly, symbionts that manipulate plant defense response to the benefit of their insect host may also affect other herbivores sharing the same plant. For instance fungal *Grosmannia clavigera* associated with *D. ponderosae* facilitate them feeding on the *Pinus banksiana* (jack bean). Feeding on plants by beetles inoculated with this fungus stimulate the increase of concentration of monoterpenes in the needles of the plant. In result, *Choristoneura pinus* (jack pine budworm, Tortricidae) feeds more, probably to compensate for decline of food quality (Colgan and Erbilgin 2011). Hence, symbiotic partner is also able to reduce food quality for its interspecific competitor.

Symbionts of insects have also impact on the levels of insects’ proteolytic enzymes (Visôtto et al. 2009), carbohydrate metabolism, enhancement of nutrient absorption (Engel et al. 2012), protein synthesis (Burnum et al. 2011), and proteases production (Rao et al. 1998). Coleopteran insects may acquire new capabilities from their symbionts via horizontal gene transfer. For example, some beetles acquired plant cell wall-degrading enzymes (PCWDE) from fungi or bacteria. For instance, β-fructofuranosidases (breaking down plant sucrose enzyme) were obtained by some Coleoptera throughout horizontal transfer, probably from bacteria. The synthesis of β-fructofuranosidases in insects’ cells (Pedezzi et al. 2014; Keeling et al. 2013) enables them to use plant sucrose more efficiently. On the other hand, Pauchet et al. (2014) indicated that wood-boring larvae (*Apriona japonica,* Cerambidae) produced arsenal of PCWDEs to the degradation hemicelluloses and celluloses in wood material. Herbivorous insects can also benefit from the presence of plant pathogen. For example, plant host responses specific for a bacterial infection may disrupt the induction of defense responses against insects (Thaler et al. 2012). In this way, activated is the signaling pathway which is antagonistic to the one activated in response to insect feeding. Consequently, the expression of genes encoding molecules that affect insect physiology is suppressed (Fig. 2b).

It is unavoidable for insect to acquire during feeding the plant material without phyllosphere microbes (both pathogens and non-pathogens), but, nonetheless, large part of non-entomopathogenic plant bacteria is killed by the alkaline gut pH, digestive enzymes, and redox potential (reactive oxygen species) or the ionic strength of the insect midgut (Vallet-Gely et al. 2009). Some evidences indicate that phyllosphere bacteria may colonize insect gut as well (Tang et al. 2012; Mason and Raffa 2014). The bacteria composition depends on plant species and genotype (Mason et al. 2015; Broderick et al. 2004). It is considered that the diversification and evolutionary success of Coleoptera have also depended on relationship with beneficial microorganisms, which have huge impact for many aspects of insect life. We are at the beginning of understanding how insect microorganisms manipulate plant response. It is important therefore to continue studies on insect- and plant-associated organisms because manipulating with symbionts and their content may be exploited to improve pest control in the future.

## Modification of plant defenses by coleopteran insect-associated bacteria

The differences in plant responses to mechanical wounding and wounding by insect feeding are mainly because of the presence of HAOEs. In addition, the application of insect oral secretions to a wound can induce a plant response similar to the one activated by herbivores attack (Lawrence et al. 2008; Erb et al. 2009). The microbes present in insect oral secretions are likely largely responsible for inducing the plant responses. The modification of plant response to insect feeding by insect-associated bacteria becomes more and more studied for coleopteran insect–plant models. Previous studies indicated that the application of oral secretions from *L. decemlineata* larvae to mechanically wounded plant tissue suppressed plant defense responses, when compared with control plants (application of water on the wounded plants) (Lawrence et al. 2007; 2008; Chung and Felton 2011). Chung et al. (2013) analyzed whether microbes in insect oral secretions could modify plant responses to benefit of the beetles. They examined antibiotic-treated and untreated *L. decemlineata* larvae. In the case of the challenge of the plant by untreated larvae, the expression of JA-dependent genes, such as polyphenol oxidase (*PPOF*/*B*) and cysteine PI, were down-regulated, while SA-dependent genes were up-regulated (*PR1, 4*). The symbiotic bacteria associated with *L. decemlineata* larvae were responsible for the down-regulation of these genes and increased *L. decemlineata* larvae performance. The neonate larvae that fed on leaves damaged by untreated larvae gained more weight than the larvae that fed on leaves damaged by antibiotic-treated larvae due to probably suppression of synthesis of plant antinutritional proteins by insect-associated microbes. Results from experiments in which bacteria isolated from *L. decemlineata* larval oral secretions were applied to wounded plants confirmed that symbionts belonging to the genera *Stenotrophomonas*, *Pseudomonas*, and *Enterobacter* are responsible for plant defense suppression. These results suggest that plant defense responses are directed against the microbes, and help to explain how the *L. decemlineata* is able to overcome plant defense responses. Therefore, microbes associated with herbivorous insects are believed to induce signaling pathways (SA and JA cross-talk) differently from the response induced by insect feeding (e.g., *L. decemlineata*), (Chung et al. 2013) shifting the plant response in the direction of SA pathway rather than JA-pathway activation.

Barr et al. (2010) assessed whether insect-associated organisms could modify the interaction between plants and insects. They used antibiotic-treated and untreated *D. virgifera virgifera* larvae and observed that untreated larvae down-regulated most plant defense genes compared with antibiotic-treated larvae and controls. The expression of the following genes was down-regulated: glutathione-*S*-transferase (responsible for detoxification of harmful substances derived from insects or bacteria), shikimate kinase (involved in synthesis of aromatic compounds, which may inhibit insect feeding and attract insect predators) (Pare and Tumlinson 1999), lipoxygenase, and lipoxygenase-related proteins (involved in the production of oxylipins and protease inhibitors) (Kessler et al. 2004). A decrease in the expression of genes encoding cinnamoyl-CoA reductase and cinnamyl alcohol dehydrogenase, which are involved in strengthening the plant cell wall by lignification, was also observed in maize. As a result, plant tissue remained palatable and digestible for insects, and larvae could easily burrow into the root tissue. In addition, the down-regulation of genes encoding glycoproteins weakened the plant cell wall (Garcia-Muniz et al. 1998).

During insect feeding, plants must coordinate the defense responses induced by wounding and HAOEs. Unfortunately, how the effectors in oral secretions modify plant defenses to benefit herbivorous insects is not fully understood. Further studies are necessary to provide deeper insights into how insect oral secretions affect plant defense responses.

## Plant microbes and their impact on plant defense responses

Microbes associated with plants may have positive, negative, or neutral effects on their hosts. The relationship between plants and microbes is usually based on mutualism. In most cases, beneficial microbes are located in the rhizosphere [plant growth-promoting rhizobacteria (PGPR), which can affect plant productivity] (Lugtenberg and Kamilova 2009) but there are also bacteria, such as endophytes that colonize the phyllosphere (Berendsen et al. 2012). The most common endophytic taxa inhabiting plant tissue are *Proteobacteria* (*Azospirillum*, *Enterobacter*, *Pantoea*, and *Pseudomonas*), *Bacteroidetes* (*Flavobacterium*), and *Firmicutes* (*Bacillus*) (McInroy and Kloepper 1995). For example, in the stem of pea plants, the most frequently observed bacteria were *Pantoea agglomerans* and *Pseudomonas fluorescens*. Less frequently observed were *Pseudomonas viridiflava* and *Bacillus megaterium* (Elvira-Recuenco and van Vuurde 2000). In addition, ten bacterial species were identified in *Jacaranda decurrens*, mostly from five genera: *Bacillus*, *Pseudomonas*, *Corynebacterium*, *Actinomyces*, and *Staphylococcus* (Carrim et al. 2006).

Endophytes are bacteria and fungi associated with plants that do not cause any apparent disease symptoms (Clay and Schardl 2002). Many endophytes enhance the growth of their hosts (Nassar et al. 2005), improve the ability of their hosts to tolerate abiotic stresses, and enhance resistance to herbivorous insects (Czeplick and Faeth 2009). Ryan et al. (2008) categorized endophytic bacteria into four groups based on their roles: (1) microbes that promote plant growth and development through the production of phytohormones (indole-3-acetic acid) (Pietr 1990) to increase the absorption of nutrients or binding of free nitrogen, (2) microbes that produce antibiotics, immunosuppressants, and bioinsecticides, (3) microbes capable of inducing plant systemic responses, and (4) microbes that improve environmental conditions through disposal of toxic chemicals (Ryan et al. 2008). Therefore, endophytes can help plants in two ways, through the antagonistic behavior toward pathogens (production of bioactive substances) and induction of plant systemic responses.

Foliar endophytes can improve plant nutrient acquisition, protect against abiotic stress (Rodriguez et al. 2009), and mediate the interaction between plants and herbivorous insects (Hartley and Gange 2009). Studies have demonstrated that some grasses are protected against herbivorous insects through vertically transmitted endophytes, resulting in the production of toxic secondary metabolites (Schardl et al. 2004; Müller and Krauss 2005). However, the presence of endophytes can also have negative effects on the natural enemies of herbivorous insects. The composition of volatiles in plants with endophytes may be different from that of plants free of endophytes (Yue et al. 2001; Jallow et al. 2008). In addition, endophytes may also mediate herbivore-induced emission of plant volatiles, resulting in the attraction of predators of herbivorous insects (Takabayashi and Dicke 1996). For example, *Pseudomonas putida* produces phenazine, which protects potatoes against soft root rots caused by *Erwinia carotovora*, whereas pyrrolnitrin synthesized by *P. fluorescens* acts against *Rhizoctonia solani* (Howell and Stipanovic 1979). However, there is very little published information regarding the protective role of plant endophytes against coleopteran species.

Some plant microbes can directly interfere with insect fitness by producing toxins. For instance *Bacillus thuringiensis* (*Bt*) produce crystal proteins acting as insecticides by forming pores in the epithelial midgut cells (Vachon et al. 2012). In addition, bacteria employ additional toxins and various effectors that interfere with insect immunity and promote infection (Nielsen-LeRoux et al. 2012). Spores of these bacteria occur in the soil. Studies have shown that these bacteria can colonize the phyllosphere, and can be taken up by the insects when they ingest plant material (Bizzarri and Bishop 2008; Mennerat et al. 2009). The toxicity of the *Bt* colonizing plant depends on bacterial strain and host plant species (Bizzarri and Bishop 2008; Monnerat et al. 2009). *Bt* toxin was used as a biopesticide to kill a range of leaf-eating insects (van Frankenhuyzen 2009), for instance, to limit harmfulness of *D. virgifera virgifera* in maize plantations in the USA. The field trials with *Bt* toxin started in 2003; however, in 2011, resistance of *D. virgifera virgifera* to *Bt* toxin was reported (Gassman et al. 2011). It was claimed that *Bt* needs a cooperation from commensal gut bacteria to be fully pathogenic, but Raymond et al. (2010) opposed to this hypothesis suggesting that *Bt* does not require assistance of other microbes for its pathogenicity. Therefore, additional studies should be done to clarify the mechanisms of pathogenicity of this bacterium to various insect species as well as *D. virgifera virgifera* resistance toward *Bt* toxin.

Information regarding the interaction among plants, insects, and bacteria is rapidly increasing as evidenced by the growing number of publications on this topic. The two-way interaction (plant–insect) had long been the subject of the research. At present, scientists start to focus rather on the three-way interaction (plant–insect–microbes). However, in the light of emerging research showing the wealth of the bacteria inhabiting the phyllosphere as well as disclosure of further details describing the plant–insect battle as being more and more complex, it can be assumed that in the future rather the four-way insect–bacteria–bacteria–plant interactions will and should be studied. Nowadays, however, the aim is a more comprehensive understanding of the role of bacteria in the interaction between plants and insects which may lead to the development of new methods of control of harmful insects populations. This will be increasingly important as more and more insects develop insecticide resistance. This aspect is of particular interest as the phenomenon of insecticide resistance in the case of beetle pests expands rapidly (Makūnas et al. 2000). The knowledge about possible contribution of insect-associated microbes in this process would be extremely important for the development of the control strategies for the protection of the most important staple food crop around the world. In addition, another future direction in research on plant–insect interaction should be to explain the impact of insects’ gut microbiota on the susceptibility of insects to pathogens (as shown previously in the case of increasing resistance toward *B. thuringiensis* toxin in *D. virgifera virgifera*). There are many original research and review articles published on the topic of microbe structural and functional diversity and the interactions between microorganisms and their plant and insect hosts (Engel and Moran 2013; Kikuchi et al. 2012; Frago et al. 2012). Our review complements what has been published so far by comprehensively reviewing the available information relevant to the biggest insect class.

### *Author contribution statement*

BW and AOS contributed to this manuscript and were involved in the drafting, preparation of models and critical revision as well as have agreed to its final content.

## Abbreviations

HAOEs: Herbivore-associated organisms and elicitors
PR: Pathogenesis-related proteins
PI: Protease inhibitor
α-AI: Alpha-amylase inhibitor
JA: Jasmonic acid
SA: Salicylic acid
ET: Ethylene
ABA: Abscisic acid
GA: Gibberellic acid
IAA: Indole-3-acetic acid
ISR: Induced systemic resistance
SAR: Systemic acquired resistance
E-64: *Trans*-epoxysuccinyl-l-leucylamido(4-guanidino)butane
AEBSF: 4-(2-Aminoethyl)benzenesulfonyl fluoride hydrochloride)
PCWDE: Plant cell wall-degrading enzymes
*Bt*: *Bacillus thuringiensis*
PPO: Polyphenol oxidase
CK: Cytokinin
PGPR: Plant growth-promoting rhizobacteria
